# COVID‐19 outbreak on the Diamond Princess Cruise Ship in February 2020

**DOI:** 10.1002/jgf2.326

**Published:** 2020-07-29

**Authors:** Yasuharu Tokuda, Tomoko Sakihama, Makoto Aoki, Kiyosu Taniguchi, Gautam A. Deshpande, Satoshi Suzuki, Sakon Uda, Kiyoshi Kurokawa

**Affiliations:** ^1^ Muribushi Okinawa Center for Teaching Hospitals Okinawa Japan; ^2^ International University of Health and Welfare Graduate School Tokyo Japan; ^3^ Sakura Seiki, Co. Tokyo Japan; ^4^ National Hospital Organization Mie National Hospital Mie Japan; ^5^ John A. Burns School of Medicine Univ of Hawaii Honolulu HI USA; ^6^ Tone Chuo Hospital Gunma Japan; ^7^ Business Breakthrough University Tokyo Japan; ^8^ National Graduate Institute for Policy Studies Tokyo Japan

The COVID‐19 outbreak on the Diamond Princess cruise ship (2666 passengers, 1045 crew; total 3711) resulted in 712 infected persons, or about 20% of the ship's population. Since the outbreak first began, thirteen deaths (case fatality rate, 1.8%) associated with the COVID‐19 outbreak on the ship have been reported. Numerous sources have suggested that quarantine measures on the Diamond Princess, which docked off Yokohama, Japan, on February 4, 2020, were inadequate to control a COVID‐19 outbreak, and allowed further spread of the virus among passengers, crew, healthcare providers, and quarantine officers.^1^, ^2^ Despite these claims, detailed information on the actual activities related to infection prevention measures undertaken on the ship remains unclear. Thus, we conducted an interviewing survey with 12 healthcare providers who participated in quarantine activities on the ship during this outbreak, identifying several salient issues regarding enacted infection control measures (Figures 1, 2, 3, 4). In addition, we provide learning points formulated as recommendations for future control of infectious disease outbreak.

**FIGURE 1:**
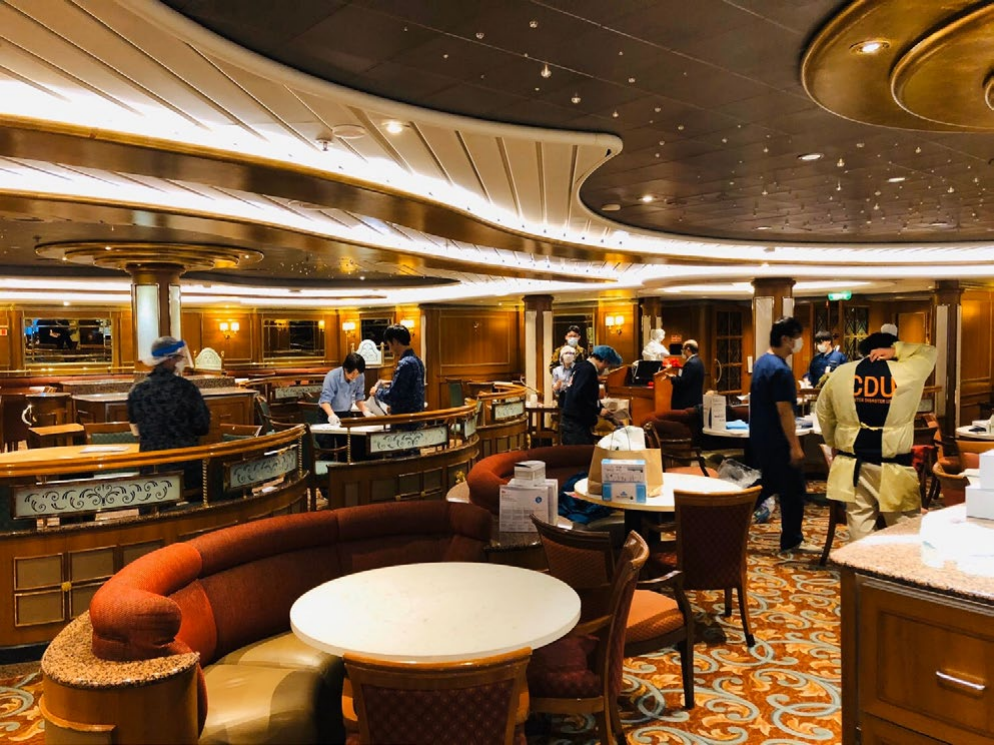
Dining room

**FIGURE 2:**
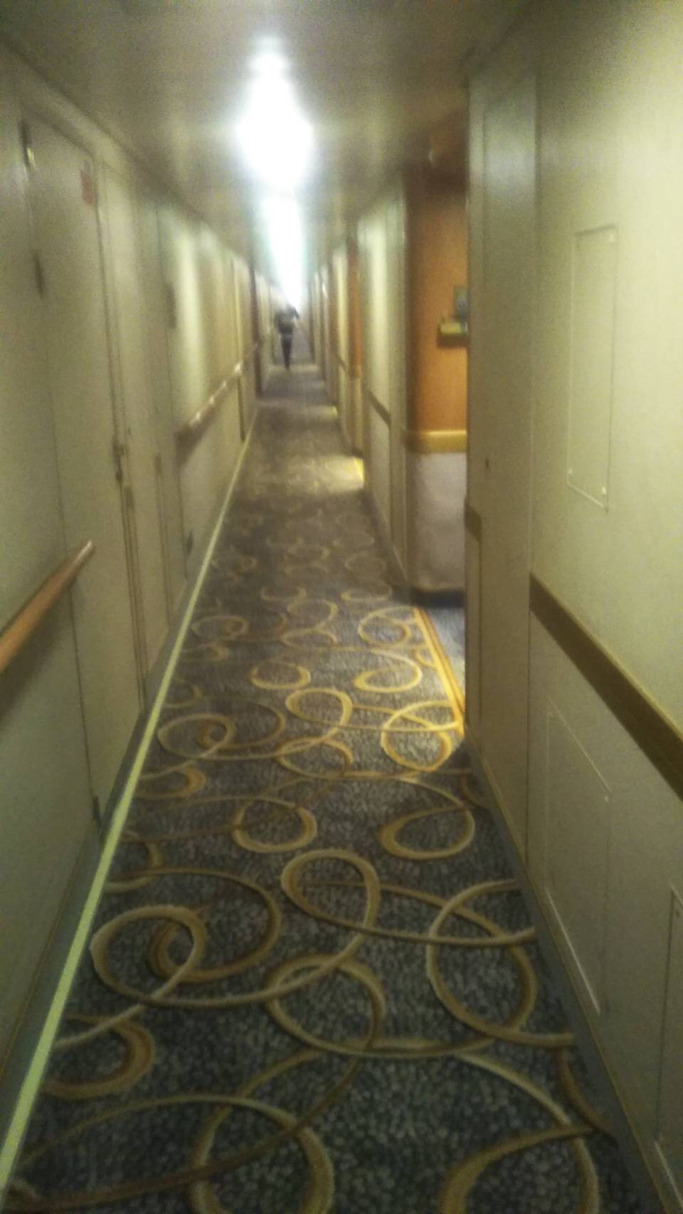
Walkway and cabins

**FIGURE 3:**
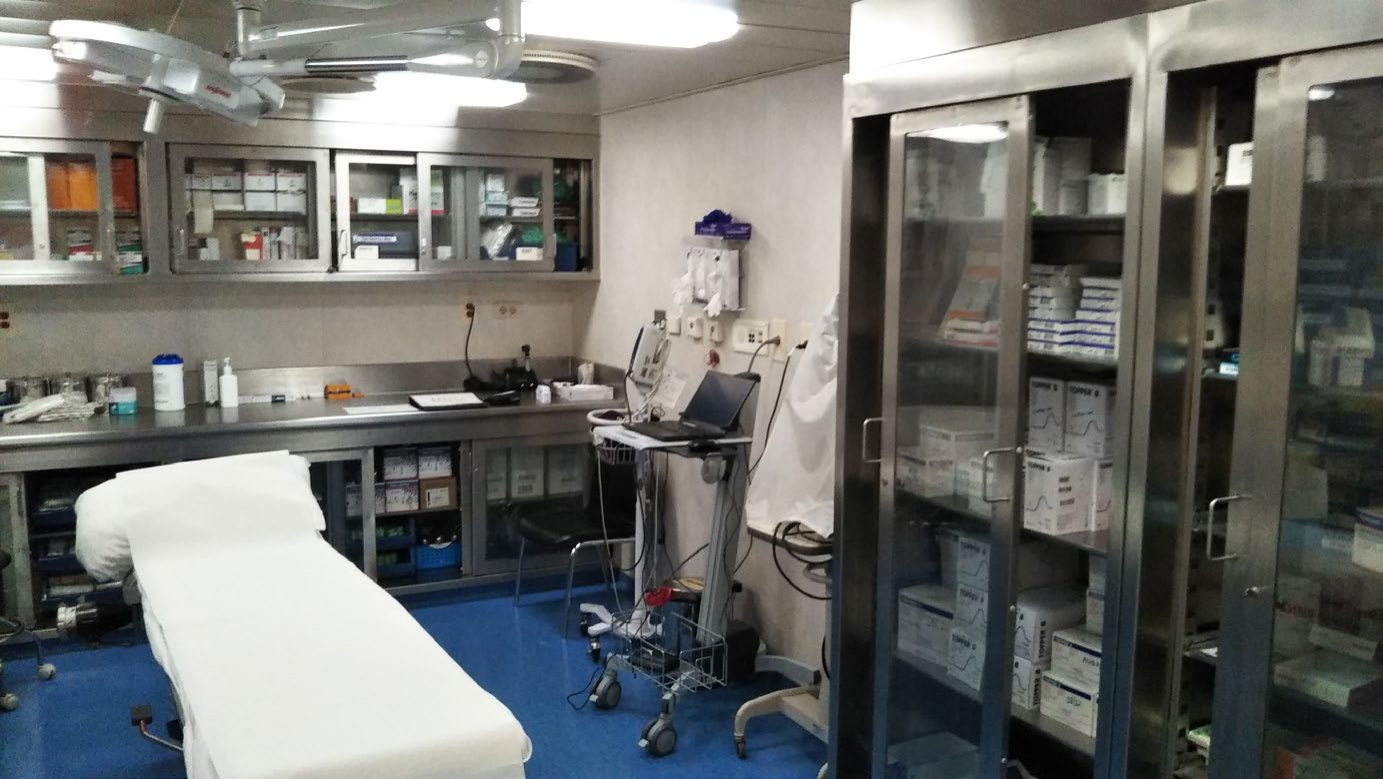
Medical room

**FIGURE 4:**
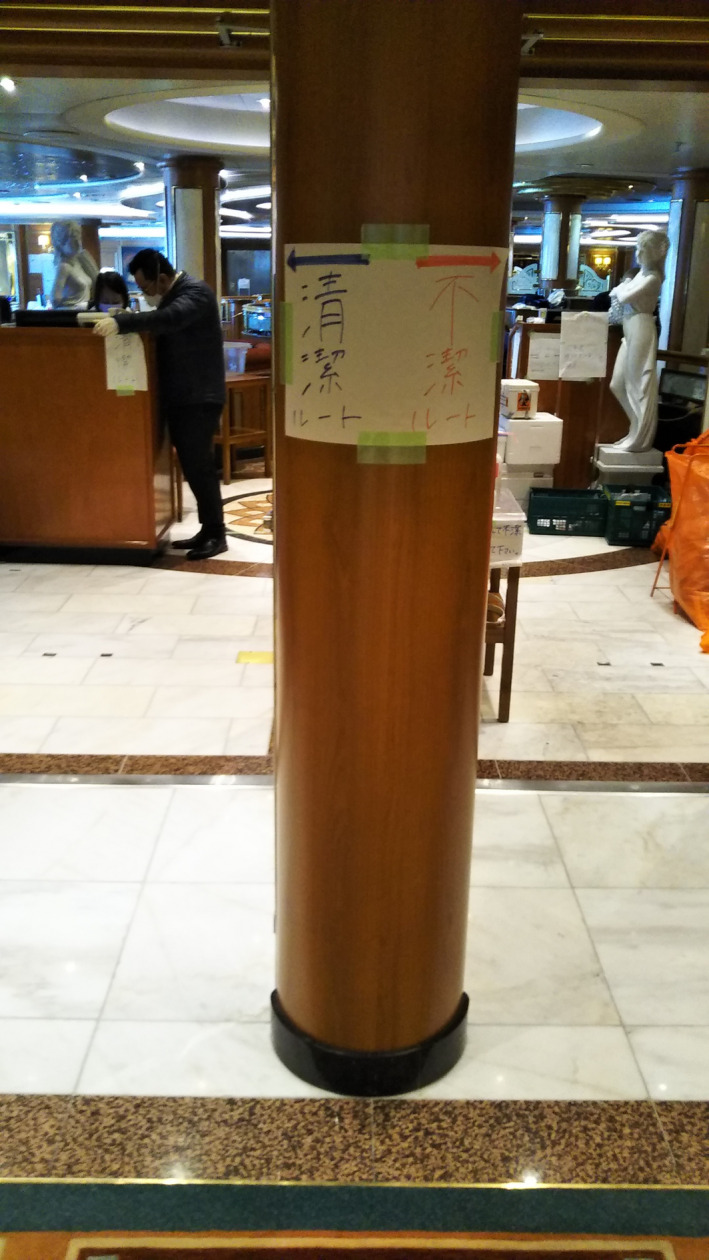
Demarcation of zoning area (red, contaminated; blue, noncontaminated)

First, crew were allowed to remain working on the ship during the quarantine, even after identification of close contact between some of these crew and the index patient, who disembarked in Hong Kong on January 25 and was subsequently found to be positive for SARS‐CoV‐2. Several crewmembers, later identified as infected, had continued to work in roles allowing for potential further spread, including providing guest services and meals to passengers during the quarantine. This may have been a potent route of continued transmission, as at least five passengers with close contact to these crewmembers subsequently developed COVID‐19 symptoms.

LEARNING POINT: Early isolation of exposed staff may be critical in stemming continued infection. Balanced with the continued operation of critical organizational functions, the availability of reserve staff or other crew relief may be important strategies in not only the ship setting, but also hospitals, nursing homes, and clinics.

Second, it was observed that many quarantine officers provided from the Ministry of Health, Welfare and Labour of the Japanese government wore surgical masks during the majority of their activities inside the ship. Subsequently, at least five officers developed COVID‐19 disease. Evidence indicates that, while hand hygiene is effective for preventing a variety of infections including influenza, wearing surgical masks alone offers little preventive benefit.^3^ Infections among multiple officers only donning surgical masks during the activity may support this evidence.

During the initial period of the quarantine, two dining rooms, which were used as administrative space, were likely to become areas for cross‐contamination between people and contaminated clothing. Most were likely to have been infected in the poorly ventilated and crowded dining room area. Passengers had been mingling together in dining rooms during two days before quarantine officers boarded the ship. In addition, clear distinctions between green and red zones were not implemented, precluding meaningful droplet and/or contact precautions in several spaces.

LEARNING POINT: In high‐risk spaces, clear demarcations of contaminated and noncontaminated areas should be implemented early. Administrative workspaces should be limited to enforced green zones. Workers should be provided with adequate personal protective equipment, including N95 mask, and protective eyewear (glasses, goggles, or face shields). Alternatively, if enforceable green zones are not possible, administrative workspaces should be placed well outside of potentially exposed areas.

Third, neither infectious disease specialists nor infection control personnel were consistently available during the entire quarantine period. Instead, Disaster Medical Assistance Team (DMAT), mostly comprised of emergency medicine providers, were asked to perform triage in front of passengers' rooms, a task reportedly difficult because of the suboptimal environment. Clinical conditions of several patients initially triaged as mild cases worsened during the period of quarantine. The DMAT members were unable to transport infected patients in a timely fashion to nearby hospitals for isolation, observation, and care. A limited number of the DMAT members needed taking care of a large number of sick people. The triage was also performed by telephone because of a large size of the ship, and it was difficult to perform precise triage.

LEARNING POINT: Identification and recruitment of relevant specialist providers, as well as ensuring their adequate availability, is critical to proper crisis management. Based on size and complexity, multiple teams of specialists may be necessary, highlighting the importance of adequate interorganizational coordination and communication. As disaster crises, by definition, may arrive in a variety of unpredictable forms, flexibility in marshaling the correct specialty resources should be anticipated.

Fourth, there were inadequate levels of cooperation, communication, and command management among multiple relevant organizations responding to the crisis. While the government was understandably unable to predict a serious infectious outbreak on a huge cruise ship with thousands of passengers, no protocol or comprehensive approach plan had been established a priori. Since both the ship and population was large (size by meters for length 290 and width 37.5; passenger cabins, 1339; crewmember cabins, 650), the ship control center was likely in confusion with tremendous amount of incoming orders from a variety of domestic organizations (multiple hospitals, academic societies, medical association, and the government), making it difficult to manage all communications effectively for disseminating standardized infection control measures throughout all people on the board. The ship control center might not be familiar with such catastrophic event. Despite the government having experienced the Fukushima Nuclear Plant Disaster 9 years previously, it is unfortunate that the level of response and resilience to unexpected disaster crises remains low.^4^

LEARNING POINT: Management of crisis events, including epidemic disease phenomena like the one posed by COVID‐19, as well as mass casualty events, requires a substantial degree of organizational planning, which should be anticipated to the utmost degree possible, with simulation activities, such as drills, conducted for possible similar events in the future. It is important to appoint a central disaster/crisis commander, define the scope of responsibilities of this role, and to collect, prioritize, and effectively announce critical information to affected individuals, relevant participating organizations, and the public. A ship control center would not be a professional for outbreak/emergency response, nor for infectious diseases. The most important outbreak response is command and control structure, so that all players are kept informed of updated information of the situation inside and what activities would be made in that day. Every outbreak response should have field coordinating meeting every day. Such a meeting among all players could share the important information, which affected their activities.

Despite the above issues, we acknowledge that passengers, ship crew, and government officers worked in earnest to contain the spread of this emerging infection, one with uncertain epidemiological characteristics, in spaces where even the most organized activities would have proven difficult. We commend them for their efforts throughout an exceptionally challenging, as well as mentally and physically exhausting, time. In the spirit of continuous improvement toward a place where high‐quality care and planning can contribute to saving lives even in the most unpredictable circumstances, we would like to share our results with the international scientific community in an effort to improve infection prevention measures and address crisis management in difficult settings.

## CONFLICT OF INTEREST

The authors have stated explicitly that there are no conflicts of interest in connection with this article.
